# Wheat spikelet detection on RGB images using deep machine learning

**DOI:** 10.18699/vjgb-26-09

**Published:** 2026-03

**Authors:** M.A. Genaev, I.D. Busov, Yu.V. Kruchinina, V.S. Kova, N.P. Goncharov

**Affiliations:** Institute of Cytology and Genetics of the Siberian Branch of the Russian Academy of Sciences, Novosibirsk, Russia Kurchatov Genomic Center of ICG SB RAS, Novosibirsk, Russia; Institute of Cytology and Genetics of the Siberian Branch of the Russian Academy of Sciences, Novosibirsk, Russia Novosibirsk State University, Novosibirsk, Russia; Institute of Cytology and Genetics of the Siberian Branch of the Russian Academy of Sciences, Novosibirsk, Russia Kurchatov Genomic Center of ICG SB RAS, Novosibirsk, Russia; Institute of Cytology and Genetics of the Siberian Branch of the Russian Academy of Sciences, Novosibirsk, Russia Kurchatov Genomic Center of ICG SB RAS, Novosibirsk, Russia; Institute of Cytology and Genetics of the Siberian Branch of the Russian Academy of Sciences, Novosibirsk, Russia Novosibirsk State University, Novosibirsk, Russia

**Keywords:** computer vision, deep learning, wheat, spike, spikelets per spike, phenotyping, object detection, компьютерное зрение, глубокое обучение, пшеница, колос, число колосков, фенотипирование, детекция объектов

## Abstract

This study addresses the challenge of automated high-throughput phenotyping of wheat spike characteristics using modern computer vision and deep learning methods. Accurate estimation of spikelet number is a key indicator of plant productivity, yet traditional manual counting approaches are labor-intensive, slow, and difficult to scale to large breeding datasets. To overcome these limitations, we propose a spikelet detection strategy based on simplified point annotations, where an expert marks only the centers of spikelets rather than drawing detailed segmentation masks or bounding boxes. This significantly reduces annotation time and lowers the overall cost of preparing training datasets for machine learning models. To determine the most effective way of utilizing such simplified annotations, three computational methods were explored: segmentation of binary masks using a U-Net architecture, density regression based on two-dimensional Gaussian distributions optimized via Kullback–Leibler divergence, and detection of fixed-size bounding regions using the YOLOv8 object detection framework. The models were evaluated on dedicated test datasets using both quantitative metrics (MAE, MAPE) and spatial localization metrics (Precision, Recall, F1 score). The results demonstrate that U-Net-based approaches provide consistently high accuracy in spikelet localization and counting while maintaining robustness to annotation imperfections. In contrast, the YOLOv8-based method showed reduced performance, likely due to the geometric mismatch between fixed-size boxes and the natural elongated shape of spikelets. Overall, the proposed methodology highlights the effectiveness of combining minimalistic point-level annotation with advanced segmentation models for automating phenotyping workflows. This approach has the potential to accelerate breeding programs, enhance the efficiency of large-scale phenotypic data collection, and support further development of robust computer-vision tools for plant science applications.

## Abstract (ru)

В работе рассматривается задача автоматизированного высокопроизводительного фенотипирования признаков колоса пшеницы с использованием современных методов компьютерного зрения и глубокого обучения. Точная оценка числа колосков является важным компонентом анализа продуктивности растения, однако традиционные методы ручной разметки и подсчета крайне трудоемки, плохо масштабируются и требуют значительных временных затрат. В исследовании предложен подход для эффективной детекции колосков, основанный на использовании упрощенной точечной разметки, при которой эксперт отмечает только центры колосков, без необходимости формировать трудоемкие пиксельные маски или ограничивающие рамки. Такая схема позволяет существенно снизить стоимость подготовки обучающей выборки и ускорить процесс аннотации. Для определения оптимального способа обработки упрощенной разметки были исследованы три метода: сегментация бинарных масок с помощью архитектуры U-Net, регрессия плотностных карт на основе двумерного нормального распределения и функции дивергенции Кульбака–Лейблера, а также детекция областей фиксированного размера с использованием модели YOLOv8. Проведено сравнение точности методов по количественным (MAE, MAPE) и пространственным метрикам (Precision, Recall, F1) на тестовых наборах изображений. Анализ результатов показал, что подходы, основанные на U-Net, обеспечивают высокую точность локализации и подсчета колосков при минимальных затратах на разметку данных, тогда как метод YOLOv8 менее устойчив к геометрической вариативности реальных объектов. Предложенный подход демонстрирует, что комбинация точечной разметки и современных моделей сегментации является эффективным инструментом для автоматизации фенотипирования, что может значительно ускорить селекционные исследования и расширить возможности высокопроизводительного анализа морфологических признаков растений

## Introduction

Population growth and local and global climate change necessitate accelerated breeding of agricultural crops, such as wheat, to increase yield and resistance to biotic and abiotic environmental factors (Efimov et al., 2024). One of the key stages of breeding is the phenotyping of spike parameters – the assessment of phenotypic traits, among which the number of spikelets in a spike is one of the most important indicators of wheat plant productivity (Afonnikov et al., 2016; Skripka et al., 2016; Maslova et al., 2018; Vahamidis et al., 2019).

The number of spikelets in a wheat spike has complex genetic control (Zhang B. et al., 2015) and is often associated with spike density, another important breeding and taxonomic trait (Vavilova et al., 2017, 2019; Savin, 2019).

Traditional methods of phenotyping spike parameters based on visual assessment by experts are slow, expensive, and subjective (Konopatskaia et al., 2016). This has stimulated the development of automated, computer vision-based solutions for high-throughput analysis (Li et al., 2017; Liu et al., 2017; Genaev et al., 2019).

Deep learning-based computer vision methods have proven effective in automating the phenotyping of agricultural plants (Artemenko et al., 2024). Existing approaches to solving the problem of counting spikelets can be divided into several types. The first type is based on object detection. For example, F. Khoroshevsky et al. (2021) used the RetinaNet architecture to detect and count spikelets directly in the field, achieving mean absolute percentage error (MAPE) values ranging from 9.2 to 11.5 %. L. Shi et al. (2023) applied the YOLOv5s model to detect the number of spikelets in images of spikes, obtaining an average absolute error (MAE) of 0.43 for the number of spikelets in a test sample of mature wheat. The second type of approach uses semantic segmentation. T. Misra et al. (2020) proposed the SpikeSegNet architecture based on U-Net, which achieved an accuracy of 95 %. However, what these methods have in common is the need for labor-intensive and costly full data annotation (using bounding boxes or pixel masks), which becomes a key limitation when scaling up research.

As an alternative to reduce annotation costs, point annotation is proposed, where the expert only needs to mark the center of the object. F. Chen et al. (2021) demonstrated that training with incomplete point annotation, where only 50 % of spikelets are annotated, results in a 6.5 % loss in accuracy (F1 drops from 84.15 to 78.65 %) compared to full annotation. Reducing the proportion of labeled objects to 10 % leads to a 16.5 % decrease in accuracy. Thus, reducing the labor costs of labeling by a factor of 10 preserves 83.5 % of the original accuracy, which confirms the promise of this approach. Another way to simplify data preparation is semi-automatic labeling (Alkhudaydi et al., 2019). R. Qiu et al. (2022) proposed a method where the initial model is trained on sparsely labeled data and then used to automatically generate labels, on which a new model is retrained.

These studies demonstrate progress in the automation of spike parameter phenotyping, but also point to a major problem: the complexity and cost of data preparation. Despite this progress, the task of developing an accurate and robust algorithm that works effectively with simplified annotation and maintains high accuracy remains relevant.

In this paper, we explore an alternative approach based on the use of simplified point annotation of only the centers of the spikes, which significantly reduces the time and cost of data preparation (Chen et al., 2021) and may be an effective and practical solution for widespread use in agricultural research.

## Materials and methods

**Biological material and data set**

**Fig. 1.**
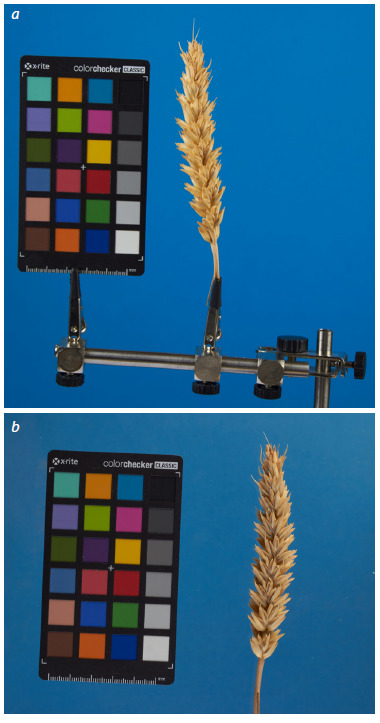
Examples of images of a wheat spike attached to a clothespin (a) and placed on a table (b).

The study used wheat spikes from the collection of N.P. Goncharov. The sample of plants included representatives of various species of diploids (2n = 2x = 14), tetraploid (2n = 4x = 14), and hexaploid (2n = 6x = 42) wheat. Images of the spikes were obtained in the laboratory according to the protocol described earlier (Genaev et al., 2018). A Canon 350D digital camera with an EF-S 18–55 mm f/3.5–5.6 lens was used for shooting. Shooting parameters: exposure 1/160, aperture 11, ISO 100, focal length 55 mm. The wheat spike was placed on a blue background next to the X-Rite Mini ColorChecker Classic color palette card (http://xritephoto.com/colorchecker-targets). Images of the spike obtained both on a clothespin and on a table were used. An example of the images used for analysis is shown in Figure 1.

In total, the dataset included 1,745 digital images of wheat spikes (82 % of images were taken on a table and 18 % on a clothespin). To evaluate the generalizability of the models, a separate holdout sample of 14 images obtained using the “on the table” protocol was used, which we did not use in either training or testing of the algorithms.

**Marking spikelets in images**

**Fig. 2.**
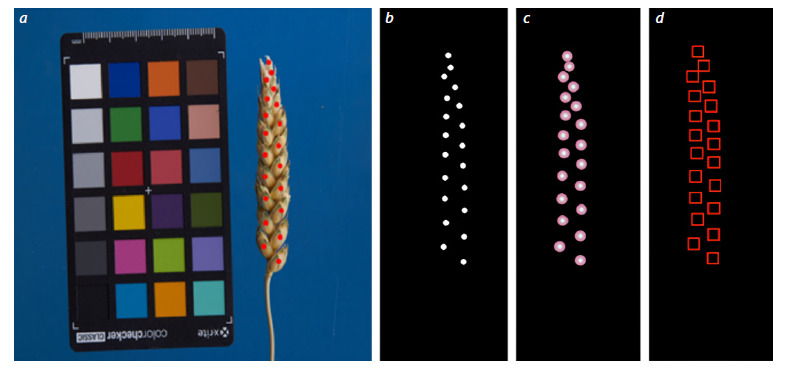
Methods for marking spikelets based on their centers, indicated manually. a – the original image of the spike with the centers of the spikelets marked with red dots; b – binary mask for spikelets in the form of circles, the centers of which coincide with the marking of spikelet centers; c – Gaussian mask, the distribution centers of which coincide with the centers of the marked spikelets, and the probability density has a radial distribution; d – a set of bounding boxes, the centers of which coincide with the centers of the spikelets.

Three types of marking were used in the study: based on binary masks, on Gaussian masks, and marking based on bounding boxes. Initially, images were marked manually in ImageJ (Schneider et al., 2012). The centers of the spikelets were marked with dots on the image, and their coordinates were saved in a separate file for each image. Based on the coordinates of the spikelet centers, three options for generating markings for machine learning were applied (Fig. 2).

Annotation in the form of binary masks (Fig. 2b). A circular area with a radius of 2 mm was generated for each spikelet. In this case, we used an image segmentation algorithm: a neural network model was trained to predict areas in the image corresponding to binary masks

Markup in the form of a Gaussian mask (Fig. 2c). It can be assumed that the centers of the spikelets marked manually do not correspond exactly to their geometric centers, but deviate randomly. We assumed that the density of this random arrangement of marks around the center has the form of a radial Gaussian distribution. Therefore, as a second marking option, a two-dimensional Gaussian distribution was generated, the center of which coincided with the marked center of the spikelet.

Bounding boxes (Fig. 2d). A square of fixed size (6 × 6 mm) was automatically generated around each manually marked spike center. The model was trained to detect these squares

**Model architectures and training**

For training purposes, the images were randomly divided into training (~60 %), validation (~20 %), and test (~20 %) samples

Two approaches were used to determine the centers of spikelets in the image using deep machine learning algorithms. The first involved segmenting the image pixels into two types: those belonging to the mask and those not belonging to the mask. For this, semantic image segmentation models based on the U-Net network (Ronneberger et al., 2015) were used. Two types of masks were used, as described above: binary and Gaussian. In the case of a binary mask, the predicted centers of the spikelets were considered to be the geometric centers of the areas corresponding to the predicted masks. In the case of a Gaussian mask, the center of the spikelet was defined as the pixel with the maximum probability within the area satisfying the condition: the ratio of the probability of each pixel to the maximum probability in a given area must not be lower than the threshold C.The U-Net network (Ronneberger et al., 2015) can use layer encoders of different architectures. In our work, several encoder options were tested: efficientnet-b3, efficientnet-b4, mit_b2, mit_b1, timm-resnest26d, timm-regnetx_032, timmres2next50, timm-gernet_m, timm-efficientnet-b4, timmefficientnet- b3 (Tan, Le, 2019; Wightman, 2019; Radosavovic et al., 2020; Zhang H. et al., 2020; Gao et al., 2021; Xie et al., 2021).

The second approach was to detect image regions bounded by squares and corresponding to the central regions of the spikelets. For this, the YOLO network architecture was used, which determines the bounding rectangle for each spikelet in the image. The YOLOv8m architecture (Redmon et al., 2016) was used for this approach.

Thus, we applied three methods to determine the centers: segmentation using the U-Net network for binary (hereinafter referred to as U-Net-BIN) and Gaussian (hereinafter referred to as U-Net-GAUSS) masks that bound the center position, and detection of the center area of the spikelet as a square using the YOLO architecture network (hereinafter referred to as YOLOv8).

Loss function type. For the algorithm with binary masks, the binary cross-entropy function (torch.nn.BCEWithLogitsLoss) was used. For Gaussian masks, the Kullback–Leibler divergence (torch.nn.KLDivLoss) was used as the loss function.

Organization of the training process. All three algorithms were trained for 500 epochs. The model weights were initialized based on pre-trained parameters obtained from the ImageNet dataset (Deng et al., 2009). The Adam algorithm was used as the parameter optimization method. During training, a set of augmentations implemented in the albumentations library (Buslaev et al., 2020) was used. The image was normalized to a size of 512×224 (Resize), then the following were applied: horizontal reflection with a probability of 0.5 (HorizontalFlip(p=0.5)); vertical reflection with a probability of 0.277 (VerticalFlip(p=0.277)); rotation by a random angle in the range −30…+30° with a probability of 0.735 (Rotate(limit=30, p=0.735)); Gaussian blur with a kernel size randomly selected in the range from 1 to 3 with a probability of 0.25 (GaussianBlur(blur_limit=(1,3), p=0.25)); addition of Gaussian noise with a probability of 0.15 (GaussNoise(p=0.15)); random brightness and contrast adjustment with a probability of 0.5 (RandomBrightnessContr ast(p=0.5)); random change in the intensity of the R, G, and B channels in the range ±15 with a probability of 0.5 (RGBShift(r_ shift_limit=15, g_shift_limit=15, b_shift_limit=15, p=0.5)); color transformations: random changes in brightness, contrast, saturation, and hue (ColorJitter(brightness=0.2, contrast=0.2, saturation=0.2, hue=0.2, p=0.703)); conversion of the image to grayscale with a probability of 0.1 (ToGray(p=0.1)).

The neural network architectures/models we investigated for identifying wheat spikelet centers depended on a number of parameters. These included: the type of encoder architecture for the U-Net network, the radius of the circular area r for binary masks, the threshold C of the ratio of the probability of a pixel belonging to the spikelet center to the maximum probability in the case of a Gaussian mask, the parameters of the optimization algorithm, and some others. Their complete list and value ranges are given in Table 1.

**Table 1.**
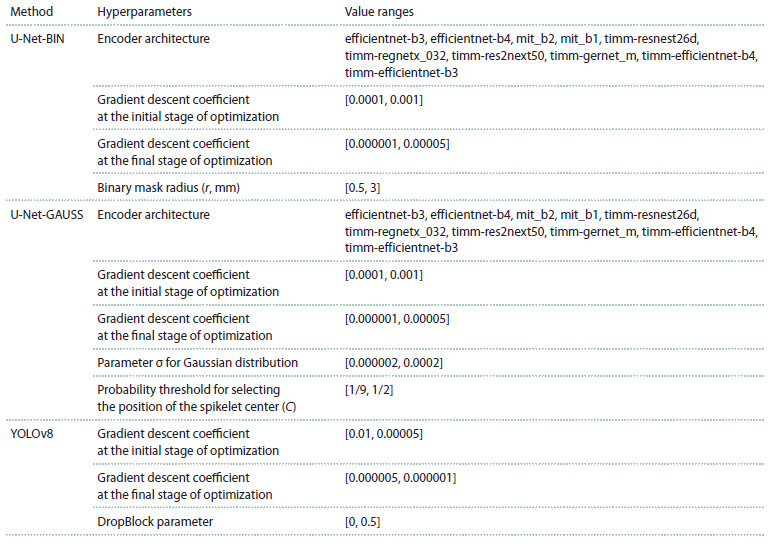
Hyperparameters used in methods for determining the position of the center of spikelets in an image

During the process, the algorithms required the selection of the most optimal parameter values from Table 1. To do this, a Bayesian optimization algorithm implemented in the Optuna library (Akiba et al., 2019) was applied using images from the validation sample. The F1 accuracy metric described below was used as the target optimization parameter.

**Algorithm accuracy metrics**

Two types of metrics were used to evaluate the effectiveness of spike center detection. Quantitative metrics evaluated the error in counting the number of spikes in the spike cluster in the image. The mean absolute error (MAE) and mean absolute percentage error (MAPE) of spike counting were evaluated as follows:

**Formula. 1.**
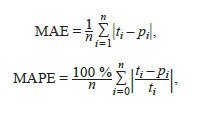
Formula 1

where ti – the true number of spikelets in the image, counted manually; pi – the number of spikelets determined based on machine learning methods; n – the number of images in the sample used to evaluate accuracy.

To evaluate the accuracy of determining the position of the center of the spikelets in the image, the metrics of precision, recall, and F1-measure were calculated.

**Formula. 2.**
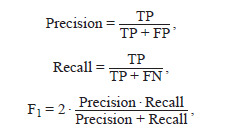
Formula 2

where TP (true positive) is the number of correct positive predictions of the position of the spikelet, FP (false positive) is the number of incorrect predictions of the position of the spikelet, and FN (false negative) is the number of false predictions of the position of the spikelet. The following rule was used to determine the TP, FP, and FN parameters (Fig. 3): the predicted center position was considered a true positive (TP) if it was within a radius of 2 mm from the true center; a false positive prediction (FP) if the spikelet center was predicted incorrectly; a false negative prediction (FN) if there libraries. The calculations were performed on a workstation with an AMD Ryzen 9 7950X processor, 64 GB of RAM, and an NVIDIA GeForce RTX 2080 Ti (11 GB) graphics accelerator.

**Fig. 3.**
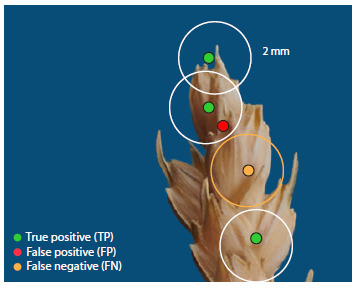
Visualization of true positives (green dots), false positives (red dots), and false negatives (yellow dots) in the calculation of spatial metrics for assessing the quality of spikelet detection.

## Results and discussion

The optimal values of hyperparameters for three models predicting the position of spikelets in the image, selected using Bayesian optimization, are listed in Table 2.

**Table 2.**
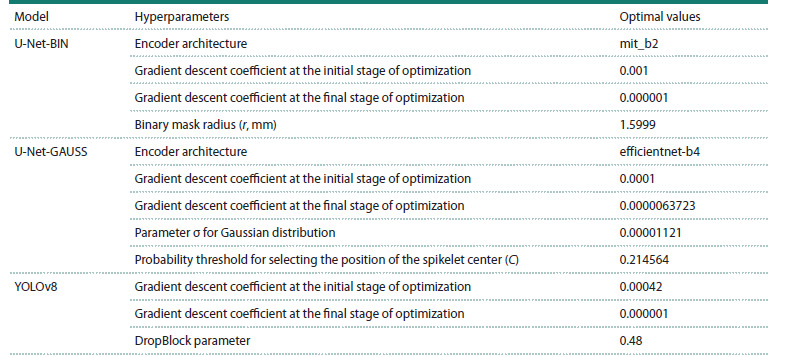
Hyperparameters for neural network models for identifying the position of spikelet centers in an image, selected using Bayesian optimization

The best encoder architecture for determining the center of the spikelet based on segmentation and a binary mask was MiT-B2 (Mix Transformer-B2) (Xie et al., 2021). This architecture combines convolutional layers with a self-attention mechanism, which allows it to simultaneously capture local features and global contextual dependencies in the image. This is especially important for the accurate positioning of small objects, such as spikelet centers

For the Gaussian mask, the EfficientNet-B4 architecture (Tan, Le, 2019) showed the best results. The model uses a compound scaling method that balances the depth, width, and resolution of the input data. This approach provides a good balance between accuracy and computational efficiency, making the architecture well suited for density distribution regression tasks.

The evaluation results on the test sample (Table 3) showed that both U-Net-based approaches significantly outperform the YOLOv8-based method.

**Table 3.**
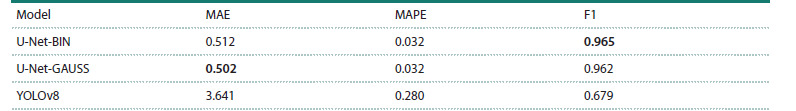
Evaluation of the effectiveness of methods for determining the position of the spikelet center on the test sample

As shown in Table 3, both approaches based on the U-Net architecture demonstrated high and comparable accuracy in terms of both quantitative (MAE ~0.5) and spatial (F1 > 0.96) metrics on the test sample. The insignificant superiority of the U-Net-GAUSS model in terms of MAE (0.502 vs. 0.512) and the U-Net-BIN model in terms of F1 (0.965 vs. 0.962) allows us to consider them equally effective for data, the distribution of which corresponds to the training sample. At the same time, the YOLOv8 model showed significantly lower results (MAE = 3.641, F1 = 0.679), which indicates its unsuitability for solving the problem in the current configuration with automatically generated square bounding boxes.

On the deferred sample (Table 4), the U-Net-BIN model showed the best accuracy, while the U-Net-GAUSS model showed lower accuracy. The largest error was observed for the YOLOv8 model.

**Table 4.**
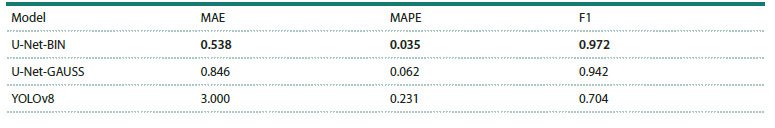
Evaluation of the effectiveness of methods for determining the position of the spikelet center on a deferred sample (n = 14)

The results on the holdout sample, collected at a different time and having a slightly different distribution, demonstrate more pronounced differences between the methods. The U-Net-BIN model maintained high accuracy, showing only a slight increase in error (MAE increased from 0.512 to 0.538), which indicates its high generalizability and reliability. In contrast, the accuracy of the U-Net-GAUSS model on the holdout sample decreased significantly (MAE = 0.846 vs. 0.502 on the test sample). This suggests that this approach may be more sensitive to data variability. The YOLOv8 model, as in the test sample, showed the worst result (MAE = 3.0), further confirming the conclusion that the selected method of generating bounding boxes is not suitable for describing the shape of spikelets.

Both proposed methods based on U-Net showed good results on the test sample. However, the model trained on binary masks demonstrated significantly better generaliza- tion ability on the holdout sample, indicating its greater reliability for working with data with a different distribution. Regression analysis of the predicted and true number of spikelets showed a strong linear relationship for U-Net models (R2 > 0.95 on the test sample), confirming the high accuracy of the count.

Measuring the inference speed on a single graphics accelerator showed that the processing time for a single image for the model trained on Gaussian masks is approximately 0.67 seconds, while for the model on binary masks it is approximately 0.64 seconds. Thus, the U-Net-BIN approach demonstrates not only higher accuracy but also slightly higher speed.

**Fig. 4.**
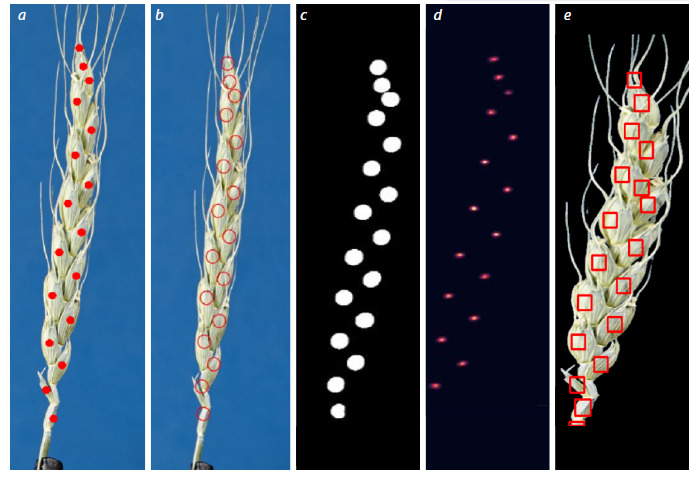
Model results. a – manually labeled centers of spikelets in a spike; b – the result of the model trained on binary masks superimposed on the actual image of the spike; c – the result of the model trained on binary masks; d – the result of the model trained on radial Gaussian distribution masks; e – the result of the model trained on bounding boxes.

The low performance of YOLOv8 is probably due to the suboptimality of automatically generated square frames for describing the elongated and curved shape of spikelets (Fig. 4). This indicates that detection methods require more careful and, possibly, manual adjustment of the markup.

## Conclusion

This study demonstrates that the use of simplified point labeling of spikelet centers in combination with modern deep learning architectures allows for accuracy comparable to the best modern methods using more complex and expensive labeling (Misra et al., 2020; Khoroshevsky et al., 2021; Zhou et al., 2021; Shi et al., 2023). The use of a simplified annotation scheme not only reduces the cost of experiments but also opens up the possibility of rapidly expanding datasets. The proposed approach can be adapted to solve other problems of counting morphological traits of plants and integrated into automated phenotyping systems to accelerate breeding programs

## Conflict of interest

The authors declare no conflict of interest.

## References

Afonnikov D.A., Genaev M.A., Doroshkov A.V., Komyshev E.G.,
Pshenichnikova T.A. Methods of high-throughput plant phenotyping
for large-scale breeding and genetic experiments. Russ J Genet.
2016;52(7):688-701. doi 10.1134/S1022795416070024

Akiba T., Sano S., Yanase T., Ohta T., Koyama M. Optuna: a nextgeneration
hyperparameter optimization framework. In: Proceedings
of the 25th ACM SIGKDD International Conference on Knowledge
Discovery & Data Mining, KDD 2019, Anchorage, AK, USA. ACM,
2019;2623-2631. doi 10.1145/3292500.3330701

Alkhudaydi T., Zhou J., De La Iglesia B. SpikeletFCN: counting spikelets
from infield wheat crop images using fully convolutional networks.
In: Artificial Intelligence and Soft Computing. ICAISC 2019.
Lecture Notes in Computer Science. Vol. 11508. Springer, Cham.,
2019;3-13. doi 10.1007/978-3-030-20912-4_1

Artemenko N.V., Epifanov R.U., Genaev M.A., Komyshev E.G.,
Kruchinina Yu.V., Koval V.S., Goncharov N.P., Afonnikov D.A.
Image-
based classification of wheat spikes by glume pubescence
using convolutional neural networks. Front Plant Sci. 2024;14:
1336192. doi 10.3389/fpls.2023.1336192

Buslaev A., Iglovikov V.I., Khvedchenya E., Parinov A., Druzhinin M.,
Kalinin A. Albumentations: fast and flexible image augmentations.
Information. 2020;11(2):125. doi 10.3390/info11020125

Chen F., Pound M.P., French A.P. Learning to localise and count with incomplete
dot-annotations. In: 2021 IEEE/CVF International Conference
on Computer Vision Workshops (ICCVW). Montreal, BC,
Canada. IEEE, 2021;1612-1620. doi 10.1109/ICCVW54120.2021.
00186

Deng J., Dong W., Socher R., Li L.J., Li K., Fei-Fei L. ImageNet:
a large-scale hierarchical image database. In: IEEE Conference on
Computer Vision and Pattern Recognition, 2009, Miami. IEEE,
2009;248-255. doi 10.1109/CVPR.2009.5206848

Efimov V.М., Rechkin D.V., Goncharov N.P. Multivariate analysis
of long-term climate data in connection with yield, earliness and
the problem of global warming. Vavilovskii Zhurnal Genetiki i Selektsii
= Vavilov J Genet
Breed. 2024;28(2):155-165. doi 10.18699/
vjgb-24-18

Gao S.-H., Cheng M.-M., Zhao K., Zhang X.-Y., Yang M.-H., Torr P.
Res2Net: a new multi-scale backbone architecture. IEEE Transactions
on Pattern Analysis and Machine Intelligence. 2021;43(2):
652-662. doi 10.1109/TPAMI.2019.2938758

Genaev M.A., Komyshev E.G., Khao F., Koval V.S., Goncharov N.P.,
Afonnikov D.A. SpikeDroidDB: an information system for annotation
of morphometric characteristics of wheat spike. Vavilovskii Zhurnal
Genetiki i Selektsii = Vavilov J Genet
Breed. 2018;22(1):132-
140. doi 10.18699/VJ18.340 (in Russian)

Genaev M.A., Komyshev E.G., Smirnov N.V., Kruchinina Y.V., Goncharov
N.P., Afonnikov D.A. Morphometry of the wheat spike by
analyzing 2D images. Agronomy. 2019;9(7):390. doi 10.3390/
agronomy9070390

Khoroshevsky F., Khoroshevsky S., Bar-Hillel A. Parts-per-object
count in agricultural images: solving phenotyping problems via a
single deep neural network. Remote Sens. 2021;13(13):2496. doi
10.3390/rs13132496

Konopatskaia I., Vavilova V., Blinov A., Goncharov N.P. Spike morphology
genes in wheat species (Triticum L.). Proc Latv Acad Sci
B Nat Exact Appl Sci. 2016;70(6):345-355. doi 10.1515/prolas-
2016-0053

Li Q., Cai J., Berger B., Okamoto M., Miklavcic S.J. Detecting spikes of
wheat plants using neural networks with Laws texture energy. Plant
Methods. 2017;13:83. doi 10.1186/s13007-017-0231-1

Liu T., Chen W., Wang Y., Wu W., Sun C., Ding J., Guo W. Rice and
wheat grain counting method and software development based on
Android system. Comput Electron Agric. 2017;141:302-309. doi
10.1016/j.compag.2017.08.011

Maslova G., Abdryayev M.R., Sharapov I.I., Sharapova Ju.A. Correlation
analysis of yield and elements of productivity of winter soft
wheat varieties in the arid conditions of steppe zone of Middle Volga
region. Izvestiya of Samara Scientific Center of the Russian Academy
of Sciences. 2018;20(2-4):680-683. doi 10.24411/1990-5378-2018-
00153 (in Russian)

Misra T., Arora A., Marwaha S., Chinnusamy V., Rao A., Jain R., Narayan
R., Ray M., Kumar S., Raju D., Ranjan R., Nigam A., Goel S.
SpikeSegNet – a deep learning approach utilizing encoder-decoder
network with hourglass for spike segmentation and counting in
wheat plant from visual imaging. Plant Methods. 2020;16:40. doi
10.1186/s13007-020-00582-9

Qiu R., He Y., Zhang M. Automatic detection and counting of wheat
spikelet using semi-automatic labeling and deep learning. Front
Plant Sci. 2022;13:872555. doi 10.3389/fpls.2022.872555

Radosavovic I., Kosaraju R.P., Girshick R., He K., Dollár P. Designing
network design spaces. In: IEEE/CVF Conference on Computer
Vision and Pattern Recognition (CVPR), 2020, Seattle, WA,
USA. IEEE, 2020;10428-10436. doi 10.1109/CVPR42600.2020.
01044

Redmon J., Divvala S., Girshick R., Farhadi A. You Only Look Once:
unified, real-time object detection. In: IEEE Conference on Computer
Vision and Pattern Recognition (CVPR), 2016, Las Vegas, NV,
USA. IEEE, 2016;779-788. doi 10.1109/CVPR.2016.91

Ronneberger O., Fischer P., Brox T. U-Net: convolutional networks
for biomedical image segmentation. In: Medical Image Computing
and Computer-Assisted Intervention – MICCAI 2015. Lecture
Notes in Computer Science. Vol. 9351. Springer, 2015;234-241. doi
10.1007/978-3-319-24574-4_28

Savin V.Yu. Determination of the force required for stripping the
wheat ear. Inzhenernyye Tekhnologii i Sistemy = Engineering Technologies
and Systems. 2019;29(3):456-466. doi 10.15507/2658-
4123.029.201903.456-466 (in Russian)

Schneider C.A., Rasband W.S., Eliceiri K.W. NIH Image to ImageJ:
25 years of image analysis. Nat Methods. 2012;9(7):671-675. doi
10.1038/nmeth.2089

Shi L., Sun J., Dang Y., Zhang S., Sun X., Xi L., Wang J. YOLOv5s-
T:
a lightweight small object detection method for wheat spikelet
counting. Agriculture. 2023;13(4):872. doi 10.3390/agriculture
13040872

Skripka O.V., Samofalov A.P., Podgornyî S.V., Gromova S.N. Productivity
and main elements of productivity of winter wheat varieties
of intensive type, bred in the All-Russian Research Institute of
Grain Crops. Dostizheniya Nauki i Tekhniki APK = Achievements of
Science and Technology in Agro-Industrial Complex. 2016;30(9):
30-32 (in Russian)

Tan M., Le Q.V. EfficientNet: rethinking model scaling for convolutional
neural networks. In: Proceedings of the 36th International
Conference on Machine Learning, ICML 2019, Long Beach, California,
USA. ICML, 2019;6105-6114

Vahamidis P., Karamanos A.J., Economou G. Grain number determination
in durum wheat as affected by drought stress: an analysis at spike
and spikelet level. Ann Appl Biol. 2019;174(2):190-208. doi 10.1111/
aab.12487

Vavilova V., Konopatskaia I., Kuznetsova A.E., Blinov A., Goncharov
N.P. Genomic characterization of DEP1 gene in wheats with
normal and compact spike shape. BMC Genetics. 2017;18 (Suppl.1):
106. doi 10.1186/s12863-017-0583-6

Vavilova V.Yu., Konopatskaia I.D., Blinov A.G., Goncharov N.P. Interspecific
polymorphism of DEP1 genes and the spike shape in
wheats. Russ J Genet. 2019;55(7):908-913. doi 10.1134/S102279
5419070147

Wightman R. PyTorch Image Models (timm). GitHub Repository. 2019.
doi 10.5281/zenodo.4414861

Xie E., Wang W., Yu Z., Anandkumar A., Alvarez J., Luo P. SegFormer:
simple and efficient design for semantic segmentation with transformers.
arXiv. 2021. doi 10.48550/arXiv.2105.15203

Zhang B., Liu X., Xu W., Chang J., Li A., Mao X., Zhang X., Jing R.
Novel function of a putative MOC1 ortholog associated with spikelet
number per spike in common wheat. Sci Rep. 2015;5(1):12211. doi
10.1038/srep12211

Zhang H., Wu C., Zhang Z., Zhu Y., Zhang Z., Lin H., Sun Y., He T.,
Mueller J., Manmatha R., Li M., Smola A. ResNeSt: split-attention
networks. arXiv. 2020. doi 10.48550/arXiv.2004.08955

Zhou H., Riche A.B., Hawkesford M.J., Whalley W.R., Atkinson B.S.,
Sturrock C.J., Mooney S.J. Determination of wheat spike and spikelet
architecture and grain traits using X-ray Computed Tomography
imaging. Plant Methods. 2021;17(1):26. doi 10.1186/s13007-021-
00726-5

